# When and How Does the Auditory Cortex Influence Subcortical Auditory Structures? New Insights About the Roles of Descending Cortical Projections

**DOI:** 10.3389/fnins.2021.690223

**Published:** 2021-08-03

**Authors:** Samira Souffi, Fernando R. Nodal, Victoria M. Bajo, Jean-Marc Edeline

**Affiliations:** ^1^Department of Integrative and Computational Neurosciences, Paris-Saclay Institute of Neuroscience (NeuroPSI), UMR CNRS 9197, Paris-Saclay University, Orsay, France; ^2^Department of Physiology, Anatomy and Genetics, Medical Sciences Division, University of Oxford, Oxford, United Kingdom

**Keywords:** auditory processing, corticofugal projections, inferior colliculus, degraded acoustic conditions, neuromodulation, frontal cortex, auditory plasticity, active listening

## Abstract

For decades, the corticofugal descending projections have been anatomically well described but their functional role remains a puzzling question. In this review, we will first describe the contributions of neuronal networks in representing communication sounds in various types of degraded acoustic conditions from the cochlear nucleus to the primary and secondary auditory cortex. In such situations, the discrimination abilities of collicular and thalamic neurons are clearly better than those of cortical neurons although the latter remain very little affected by degraded acoustic conditions. Second, we will report the functional effects resulting from activating or inactivating corticofugal projections on functional properties of subcortical neurons. In general, modest effects have been observed in anesthetized and in awake, passively listening, animals. In contrast, in behavioral tasks including challenging conditions, behavioral performance was severely reduced by removing or transiently silencing the corticofugal descending projections. This suggests that the discriminative abilities of subcortical neurons may be sufficient in many acoustic situations. It is only in particularly challenging situations, either due to the task difficulties and/or to the degraded acoustic conditions that the corticofugal descending connections bring additional abilities. Here, we propose that it is both the top-down influences from the prefrontal cortex, and those from the neuromodulatory systems, which allow the cortical descending projections to impact behavioral performance in reshaping the functional circuitry of subcortical structures. We aim at proposing potential scenarios to explain how, and under which circumstances, these projections impact on subcortical processing and on behavioral responses.

## Introduction

The auditory cortex has been viewed as the ultimate step in processing the rich acoustic stream constantly reaching our ears and also as a key structure in cognitive tasks involving auditory stimuli (Weinberger and Diamond, 1987; Edeline, 1999; Weinberger, 2004; Ohl and Scheich, 2005; Fritz et al., 2007). Indeed, the plasticity of auditory cortex network has been described in many situations ranging from frequency discrimination (Edeline and Weinberger, 1993; Edeline et al., 1993; Fritz et al., 2003, 2005) or spatial discrimination tasks (Lee and Middlebrooks, 2011; Wood et al., 2019) to pitch extraction (Bizley et al., 2013), attentional tasks (Otazu et al., 2009), selective attention (Wittekindt et al., 2014), and predictive coding (Malmierca et al., 2015).

Besides its role in cognitive functions, several recent studies performed on different species have promoted the idea that auditory cortex is also a key structure in building noise-invariant representations of communication sounds (Narayan et al., 2007; Carruthers et al., 2013, 2015; Rabinowitz et al., 2013; Schneider and Woolley, 2013; Mesgarani et al., 2014; Ni et al., 2017; Aushana et al., 2018; Beetz et al., 2018; Town et al., 2018; Souffi et al., 2020). For example, the cortical responses to conspecific vocalizations, and their discriminations by cortical neurons were largely preserved during various types of acoustic alterations performed in the spectral and temporal domain (Souffi et al., 2020).

In this review, we propose new roles of descending cortical projections reaching the auditory thalamus and the inferior colliculus. These two subcortical structures receive the dominant part of the corticofugal inputs and had been explored in a large number of species and under different listening conditions. Therefore, we will focus on the specific effects mediated by those circuits without forgetting that the effects of the cortical descending projections can also modify earlier relay stations. We will describe studies from different animal species (mice, rats, guinea pigs, ferrets, bats, and birds). While the descending cortical projections in the auditory system are potentially equivalent in all species, the frontal circuitry could strongly vary between species making generalization of results more difficult.

In the present review, we will first describe the extent to which cortical neurons robustly code the representation of target stimuli in acoustically challenging conditions. Next, we will examine data suggesting that noise-invariant representations do also exist in subcortical auditory structures. In the last sections of the review, we will point out that, despite numerous experiments which aimed at describing the influence of corticofugal connections at the thalamic and collicular level, it is only the use of cell-targeted activation/inactivation methodologies combined with behavioral tasks that have recently unraveled whether the auditory cortex impacts on subcortical processing in challenging conditions.

## Evidence for Noise-Invariant Representations in Auditory Cortex

Our ears are constantly bombarded by a complex sound mixture, which generates challenging acoustic conditions for speech understanding. These degraded acoustic conditions can be the presence of reverberations, for example created by the shape, size, and objects in the room in closed spaces, the presence of concomitant sound sources with the particular case of the “cocktail party” noise where a target source has to be segregated from other competing sounds (e.g., see Narayan et al., 2007) but also particular environmental conditions that can attenuate specific frequencies from the signal spectra (Mesgarani et al., 2014; Fuglsang et al., 2017; Bidelman et al., 2018). All these factors lead to difficulties in perceiving target sounds such as speech, communication sounds and music in normal-hearing subjects, but cause even more difficulties for subjects with mild to moderate hearing loss, and are very penalizing for subjects with cochlear implants, a neuroprosthetic device which restores hearing in people suffering from profound deafness. Note also that for patients with cochlear implants, the descending cortical projections to the thalamus and to the inferior colliculus are preserved but the indirect cortical modulation to the auditory periphery is lacking.

Understanding what are the spectro-temporal acoustic cues used by human subjects necessary for auditory perception in challenging conditions and the neuronal mechanisms allowing the auditory system to extract relevant cues for discriminating sounds in those acoustic conditions are major aims in psychoacoustic and auditory neuroscience.

Over the last two decades, most of the studies describing the physiological consequences of adding noise on the neuronal responses to target stimuli have been performed at the level of the primary auditory cortex (A1). In their initial study, Nagarajan et al. (2002) reported that white noise addition reduced auditory responses to conspecific communication sounds (marmoset calls) only at a 0 dB signal to noise ratio (SNR), the lowest SNR tested. This study also pointed out that cortical neurons are particularly robust to spectral degradations since there was little change in evoked responses at presentation of vocoded vocalizations [an artificial signal-processing distortion that remove the spectral content and the frequency modulation (FM) cues but partially preserved the amplitude modulation (AM) cues], even in response to only 2-band vocoded vocalizations. In contrast, temporal-envelope degradations strongly reduced the evoked firing rate and the neural synchronization to the vocalization envelope. Importantly, bandpass filtering the vocalizations between 2-30 Hz did not reduce the firing rate and neural synchronization to the vocalization envelope. Similarly, subsequent studies did not find much alterations of cortical responses for speech-like sounds presented in noise: for example, Shetake et al. (2011) in rats did not find significant reduction in neural discrimination using an index of neuronal population performance at a +12 dB SNR; the neural performance fell close to the chance level only at −12 dB SNR, the lowest SNR tested. In the field L in birds (homologous to primary auditory cortex), the neural discrimination performance was maintained down to a +5 dB SNR (Narayan et al., 2007).

Recent studies in guinea pigs have confirmed that the responses of auditory cortex neurons are particularly resistant to spectral degradations of communication sounds (such as vocoded vocalizations, e.g., Souffi et al., 2020), even in the presence of masking noise (Aushana et al., 2018). At the level of small cortical populations (2–16 simultaneous recordings), the ability to discriminate between conspecific vocalizations remained almost intact despite strong spectral alterations (Aushana et al., 2018; Souffi et al., 2020).

However, analyzing in more detail the responses of individual recordings across several signal-to-noise ratios revealed strikingly different categories (Ni et al., 2017: marmoset; Souffi et al., 2021: guinea pigs), which ranged from neuronal responses robust to noise and specific to target stimuli, to neuronal responses sensitive to noise and specific to masking noises. In fact, the initial results of Bar-Yosef and Nelken (2007) in the cat primary auditory cortex have already pointed out that some cortical neurons can be more specific to the background noise than to the actual communication sounds. In addition, context seems to be important too, and neurons assigned to a particular category can change category depending on the type of noise, indicating that different types of masking noise activate different subpopulations of neurons in the auditory cortex and subcortical auditory structures (Ni et al., 2017; Souffi et al., 2021).

Several hypotheses have been formulated to account for the performance of auditory cortex neurons in detecting target stimuli in masking noise. For example, it was proposed that noise tolerance is correlated with adaptation to the stimulus statistics, which is more pronounced at the cortical than at the subcortical level in ferrets (Rabinowitz et al., 2013). A dynamic model of synaptic depression was also suggested as a potential mechanism for robust speech representation in the human auditory cortex (Mesgarani et al., 2014). Alternatively, a simple feedforward inhibition circuit operating in a sparse coding scheme was viewed as a mechanism to explain background-invariant responses detected for a population of neurons in the zebra finch secondary auditory cortex (Schneider and Woolley, 2013).

As we will see below, it is important to determine whether these mechanisms only operate at cortical level or whether they are general mechanisms operating at all the levels of the central auditory system.

## Subcortical Implications in Building Noise-Invariant Representations

Compared with the large literature focused on the auditory cortex, only a few studies have described the resistance to noise of subcortical neurons. Nonetheless, a direct comparison between the consequences of acoustic degradation in different structures is the most straightforward way for dissecting where invariant representations emerged. At the thalamic level, a massive reduction in firing rate and temporal reliability of evoked responses was reported in rats during the noise condition when target stimuli and background noise were at the same intensity level (0 dB SNR, Martin et al., 2004). In the avian auditory system, Schneider and Woolley (2013) described the emergence of noise-invariant responses for a subset of cells (the broad spike cells) of a secondary auditory area (area NCM), whereas neurons in the field L and the mesencephalicus lateralis dorsalis (homologous of the primary auditory cortex and inferior colliculus, respectively) show background-corrupted responses. They proposed that a sparse coding scheme (in the sense that neurons show less driven response to the same stimulus and respond only to a small subset of the stimuli) operating within the area NCM allows the emergence of this noise-invariant representation. Note that, in rats, such a sparse representation already exists as early as A1 (Hromádka et al., 2008).

Noise-invariant representations were also reported in A1 of anesthetized ferrets (Rabinowitz et al., 2013). This study suggested a progressive emergence of noise-invariant responses from the auditory nerve to the inferior colliculus (IC) and to A1, and proposed the adaptation to the noise statistics as a key mechanism to account for the noise-invariant representation in A1. However, Lohse et al. (2020) in mice have recently challenged this view. Indeed, they showed that collicular, thalamic and cortical neurons display similar contrast gain control with the slowest time constants in A1 and importantly, the silencing of auditory cortex, did not affect the contrast gain control capacity of neurons in the inferior colliculus or in the medial geniculate body (MGB). Previous studies have already shown adaptation to stimulus intensity of subcortical neurons. First, adaptations of IC neurons to the average stimulus intensity, stimulus variance and bimodality have already been described in guinea pigs with a temporal decay of about 160 ms at 75 dB sound pressure level (SPL, Dean et al., 2005, 2008). Second, adaptation to the noise statistics shifted the temporal modulation function (TMF) of IC neurons to slower modulations, sometimes transforming band-pass TMF to low pass TMF in about 200 ms of noise presentation (gerbils: Lesica and Grothe, 2008).

In fact, Nelken et al. (1999) in cats have previously shown that the addition of low intensity sounds interrupts the phase locking of A1 neurons to the envelope of slowly fluctuating noise (about 10 Hz). This phenomenon has been called “locking suppression.” Moreover, the high sensitivity of this suppression, occurring at intensities lower than the neuron’s threshold (at −15 or −35 dB SNR), seems to be a marked phenomenon at the cortical level, present for only about half of the neurons of the MGB and absent at the level of the IC. The conclusion is that, although the detection of pure tones in fluctuating noise is possible from the IC, the segregation between the representation of sound as a perceptual object separate from noise is more explicit/complete at the cortical level. It should be noted that intracellular recordings did not reveal a particular role of cortical inhibition in the phenomenon of “locking suppression,” it is already detected in the excitatory inputs received by cortical neurons (cats: Las et al., 2005; rats: Hershenhoren and Nelken, 2017).

**FIGURE 1:**
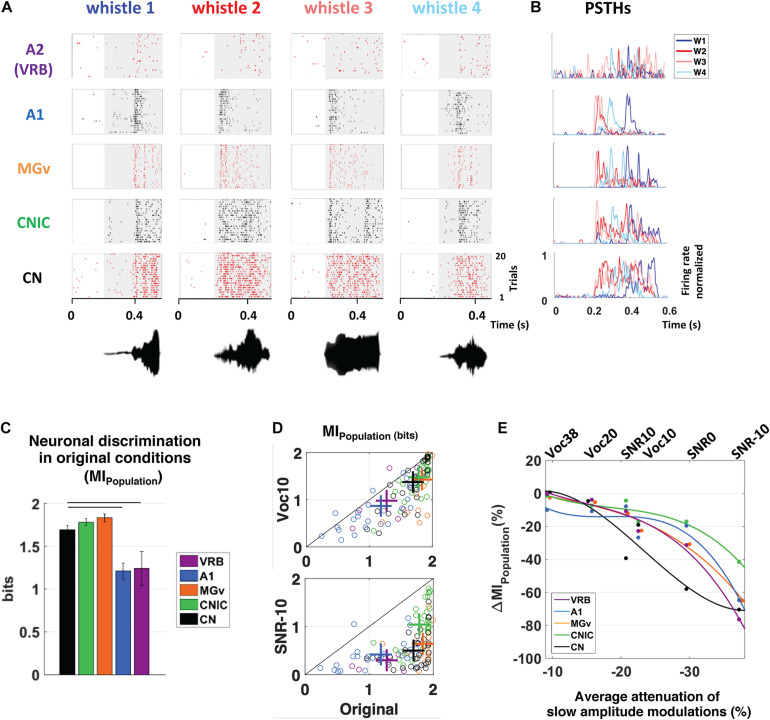
Subcortical neurons better discriminate the vocalizations in quiet as well as in degraded conditions and alterations of slow amplitude modulations are crucial cues for explaining the decrease in discrimination performance at the subcortical and cortical levels. **(A)** From bottom to top, raster plots presenting the neuronal responses recorded in CN, CNIC, MGv, A1, and VRB. Each dot represents an action potential and each line the presentation of one of four original whistles. The gray areas correspond to the evoked activity. The waveforms of the four original whistles are displayed under the raster plots. **(B)** Peristimulus time histograms (PSTHs) of the neuronal responses presented in **(A)**. For all neuronal recordings, the four PSTHs corresponding to the four original whistles have been overlayed. **(C)** The mean values of the neuronal discrimination at the population level (MI_Population_, bits) are presented for populations of 9 simultaneous multiunit recordings obtained with the four original vocalizations in CN (in black), CNIC (in green), MGv (in orange), A1 (in blue), and VRB (in purple). Error bars represent the SE of the mean and horizontal black lines represent the statistically significant differences. Note that, all the subcortical structures discriminate better the original vocalizations than cortical areas. **(D)** Scattergrams showing the modest decrease in MI_Population_ (bits) with the most severe vocoded condition (Voc10, top panel) compared to the strong decrease with the most severe noisy condition (SNR-10, bottom panel). Each cross represents the mean MI_Population_ obtained in degraded and original conditions. **(E)** Percentage of alterations in neuronal population discrimination abilities (ΔMI_Population_) as a function of the alterations in slow amplitude modulations induced by vocoding (Voc38, Voc20, and Voc10) or by the addition of stationary noise (SNR10, SNR0, and SNR-10). Each dot represents neuronal data (ΔMI_Population_) in CN (in black), CNIC (in green), MGv (in orange), A1 (in blue) and VRB (in purple). Polynomial curves fitting all acoustic conditions have been generated (color lines). In all conditions (vocoding or noise), there is a limit of AM reduction from which the ΔMI_Population_ decreases in cortical and subcortical structures. Thus, the reduction of slow AM cues is one of the factors explaining the neuronal discrimination performance at the subcortical and cortical levels. Modified from Souffi et al. (2020). CN, cochlear nucleus; CNIC, central nucleus of the inferior colliculus; MGv, ventral division of the medial geniculate nucleus; VRB, ventrorostral belt (secondary auditory cortex).

**FIGURE 2:**
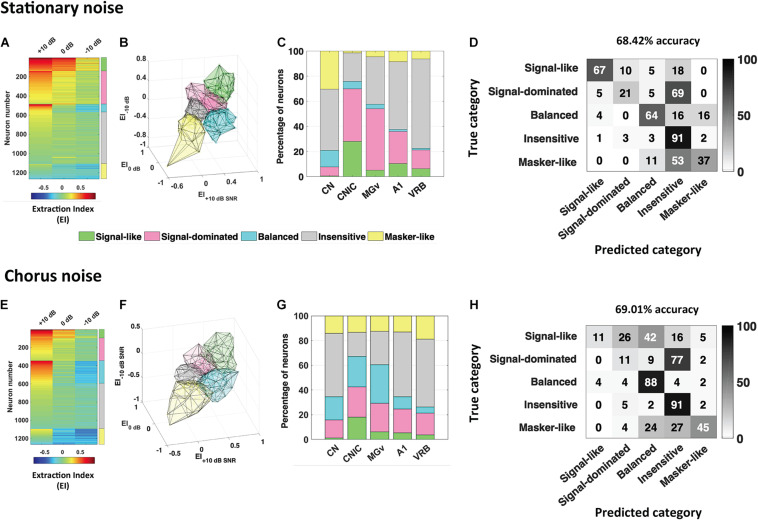
Robustness to noise is a distributed and predictable property in the whole auditory system. The extraction index (EI) quantifies to what extent the evoked response at a given SNR is similar to the response to vocalizations in quiet or to noise alone. **(A)** Each row corresponds to the extraction Index (EI) profile of a given neuronal recording obtained in the five auditory structures (CN, CNIC, MGv, A1, and VRB) in stationary noise with a color code from blue to red when progressing from low to high EI values. The color coded column on the right, delineate the identity for the five categories of responses found, “signal-like” in green, “signal-dominated” in pink, “balanced” in turquoise, “insensitive” in gray, and “masker-like” in yellow. **(B,C)** 3D representation of the five categories in stationary noise **(B)**, and proportion of each category in the five auditory structures from CN to VRB **(C)**. **(D)** Confusion matrix obtained with descriptors extracted from pure tone responses, signal alone and stationary noise alone responses. Each row corresponds to a true category and each column corresponds to a predicted category. The numbers in the confusion matrix correspond to the percentage of recordings of a given true category which have been predicted to belong to a given predicted category. Around 70% of accuracy (68.42%) was reached with these descriptors. **(E–H)** Same representations as in **(A–D)** for the responses collected in the chorus noise. Modified from Souffi et al. (2021).

From recordings obtained in anesthetized guinea pigs in the cochlear nucleus, inferior colliculus, auditory thalamus, A1 and a non-primary auditory cortex, Souffi et al. (2020) reported that higher discrimination performance and more accurate representations in degraded acoustic conditions (presence of masking noise or vocoding) were found in IC and MGB; cortical representations, although less accurate as the subcortical ones, were barely affected under these degraded conditions (Figure 1, modified from Souffi et al., 2020). Furthermore, when neuronal responses in noise were classified among a continuum in five categories from the most robust to noise (signal-like responses) to the most sensitive to noise (masker-like responses, representing accurately the masking noise), it was found, in two noise types, that these categories were distributed in the whole auditory system, with higher proportions of robust responses in inferior colliculus and thalamus (Figure 2, modified from Souffi et al., 2021). In addition, the responses to the signal alone and to the noise alone allowed the assignment of a given recording to one of five categories to be predicted up to 70%. A link between inferior colliculus activity and behavior was pointed out in two studies showing that a tone-versus-noise discrimination task modulates the neuronal activity as early as the inferior colliculus (Slee and David, 2015; Shaheen et al., 2020). In the first one in ferrets, it was found that in the active condition, collicular responses to reference sounds were mostly suppressed and this effect was frequency-dependent with lower suppression when the target frequency was away to the Best Frequency (BF) of the neuron than when was closer. The second study quantified the neuronal discriminability in a tone-masking noise task (0 dB SNR) of IC neurons in marmoset (non-lemniscal IC: dorsal and external cortices, and lemniscal IC: central nucleus) and indicated that non-lemniscal IC neurons enhanced their neuronal discriminability in active condition whereas lemniscal IC neurons did not.

All the results together suggest that noise-invariant representations emerge very early in the auditory system under conditions of anesthetized or awake passive listening, without necessarily the involvement of cortical activity (Lohse et al., 2020).

## Effects of the Corticofugal Descending Projections

A myriad of anatomical studies have described in great detail the corticofugal projections originating from auditory cortex reaching the different subcortical relays (for reviews see Winer, 2006; Winer and Lee, 2007; Malmierca and Ryugo, 2011), but only a limited set of studies have reported the physiological effects of these projections. In this review, we focus on the descending cortical projections to the thalamus and the inferior colliculus but it should be kept in mind that descending cortical projections have been anatomically described in the dorsal cochlear nucleus (Jacomme et al., 2003). Also, the activity of the auditory nerve and the cochlea could be modulated via the olivocochlear neurons (Aedo et al., 2016; for reviews: Terreros and Delano, 2015; Elgueda and Delano, 2020) that receive direct projections from the auditory cortex (rats: Mulders and Robertson, 2000; Doucet et al., 2002; guinea pig: Coomes and Schofield, 2004; Brown et al., 2013) and the inferior colliculus (Thompson and Thompson, 1993).

For the purpose of the present review, it is particularly important to distinguish the conditions during which these effects have been reported. Some of these studies performed in anesthetized animals have either activated or inactivated auditory cortex neurons and looked for the physiological consequences on the neuronal responses collected in subcortical auditory structures. Other studies, performed in awake behaving animals, have looked at the consequence of silencing the auditory cortex on the animal behavioral performance.

### Auditory Cortical Manipulations in Anesthetized Animals

The rational of the electrophysiological experiments performed in anesthetized animals was simply to record in subcortical structures during either inactivation or electrical activation of the auditory cortex. The initial topography of the corticocollicular pathway has been described in cats by Anderson et al. (1980) combining recordings in the primary auditory cortex (A1), the anterior auditory field (AAF) and the secondary auditory cortex (AII) with anterograde (3H^∗^Leucine) tracer injections and showing labeled terminals in IC, including the central nucleus where the changes in position of the labeling agreed with the tonotopic axes of the central nucleus of the IC (CNIC) and the tuning frequency of the neurons recorded at the injection sites. The glutamatergic nature of this pathway was suggested by Feliciano and Potashner (1995) after ablation of the auditory cortex in guinea pigs and determination of the uptake and release of radioactive Aspartate in the inferior colliculus. Initial experiments in cats have silenced the entire auditory cortex by cooling and have reported both excitatory and inhibitory effects on responses of auditory thalamus neurons (Ryugo and Weinberger, 1976) and in the inferior colliculus. In many cases, “On” responses were unaffected whereas long latencies responses were largely reduced (see also in rats, Cotillon and Edeline, 2000). A study in cats sampling neurons in the different MGB anatomical subdivisions (Villa et al., 1991) revealed that the increases in signal-to-noise ratio (evoked divided by spontaneous firing rate) often result from a larger decrease in spontaneous than in evoked activity. Subsequent studies using pharmacological inactivation of auditory cortex by muscimol (a long-lasting GABA_A_ agonist) or lidocaine (a local anesthetic acting on sodium channels), have reported that cortical inactivation reduced auditory responses in the ventral tonotopic lemniscal division of MGB (MGv) and in the inferior colliculus with a larger (60 vs. 34%) and faster (11 vs. 31 min) reduction for thalamic neurons than for collicular neurons (mustached bat, Zhang and Suga, 1997; Zhang et al., 1997). The effects of stimulating or blocking the activity of the auditory cortex while recording collicular neurons have been studied in different species. For example, Syka and Popelar (1984) in rats showed that most IC neurons, mainly located in the dorsal and caudal IC, reacted with a short excitation (3–15 ms) followed by inhibition lasting 30–150 ms or just inhibition after electrical stimulation of the auditory cortex (bipolar electrodes, single pulses, duration 0.2 ms, current 0.2–1.5 mA). Similar approach was used by Torterolo et al. (1998) with electrical stimulation in the guinea pig auditory cortex while recordings were performed in the IC neurons, observing differential effects on spontaneous and driven activity and different latencies depending on whether the recording was ipsilateral or contralateral to the stimulated cortex. Jen et al. (1998) recorded neurons in the CNIC of the big brown bat while blocking with lidocaine or electrically stimulating the auditory cortex. They showed corticofugal facilitation or inhibition, with longer latencies with inhibition. The cortical effect was most effective when it was combined with sounds of low intensity. The effects of phasic electrical stimulation of auditory cortex have pointed out the view that cortico-thalamic projections have an excitatory influence on thalamic activity. In guinea pigs, auditory cortex stimulation facilitated tone-evoked responses for more than 2/3 of the MGv neurons, especially when the BFs of the cortical and thalamic recordings were similar (He et al., 2002). Surprisingly, a similar cortical activation tended to induce inhibitory effects in the non-lemniscal divisions of the auditory thalamus (He, 2003), potentially due to the activation of GABAergic neurons from the thalamic reticular nucleus (Cotillon and Edeline, 2000) or from the IC (cats: Winer et al., 1996). Subsequent intracellular studies have confirmed this differential effect: depolarizations of MGB neurons in guinea pigs were only observed in the lemniscal division whereas hyperpolarizations were only observed in non-lemniscal MGB neurons (Yu et al., 2004). These changes in membrane polarizations contribute to a differential change in the acoustic responses of MGB cells (Xiong et al., 2004). In addition, they also pointed out that stimulation of the auditory cortex can modulate evoked responses in the auditory sector of the reticular nucleus and also promote a more tonic mode of discharge (Xu et al., 2007). It was speculated that the systematic selectivity of facilitation and inhibition over the lemniscal and non-lemniscal MGB is related to the attention shift within the auditory modality and across the sensory modalities (Yu et al., 2004).

The techniques used in these initial studies had obvious limitations. Besides the risks of non-specific effects (such as lowering the blood temperature during cortical cooling), the main consequence of global inactivation of the whole auditory cortex is removing its input onto corticofugal targets, including MGB and IC cells, but also onto higher cortical areas. Likewise, cortical electrical stimulation can trigger neuronal discharge in subcortical cells by both orthodromic and antidromic activation. In addition, global electrical activation or chemical inactivation obviously affects all descending projections originating from the auditory cortex, not only those reaching the subcortical structure under investigation (the MGB or the inferior colliculus). To circumvent these limitations, optogenetic tools have been used in most recent studies, to transiently silence, or activate, auditory cortex neurons in anesthetized and awake animal.

### Modulation of Cortical Projections by Optogenetic Techniques

As described in the first part of this review, some studies have suggested that there was a difference in neuronal adaptation to noise between cortical and subcortical structures (Rabinowitz et al., 2013). A more recent study in mice (Lohse et al., 2020) has reported that the contrast gain control was robust in A1, MGv and CNIC. In these experiments, the degree of adaptation to high (40 dB) or low (20 dB) contrast to dynamic random chords (DRC) was evaluated in MGv and CNIC during the silencing of cortical neurons (by activating inhibitory GABA interneurons). The contrast gain control was unchanged during cortical silencing both in anesthetized and awake mice at collicular level and in anesthetized animals at thalamic level, which clearly points out that subcortical neurons can exhibit contrast adaptation via intrinsic, cortical-independent mechanisms. Interestingly, cortical silencing had no effect on the shape of the spectro-temporal receptive fields (STRFs, i.e., BF value, spectral and temporal bandwidth, value of the largest weight in the kernel) both in MGv and CNIC. When the cortex was silenced, it is also interesting to note that (i) the reliability of responses to DRC was even increased in the MGv and in the CNIC of awake mice and that (ii) subcortical neurons were better described by a linear model than when the cortex was normally operating, as if the cortical inputs decrease the reliability and the linearity of MGv and CNIC neurons. Interestingly, in anesthetized or awake passively listening animals, the corticofugal projections did not contribute to the contrast adaptation observed in the MGv and CNIC.

However, and as it is the case with cortical cooling, one can consider that silencing the whole auditory cortex does not mimic a physiological situation. The corticofugal projections are topographically organized: Anterograde tracing studies have shown that the location of the terminal fields in the CNIC varies topographically with the location of the injection sites in A1 (rats: Saldaña et al., 1996; gerbils: Bajo and Moore, 2005; ferrets: Bajo et al., 2007, Figure 3A). Injecting tracers at two locations in ferret A1, where neurons were tuned to different frequencies, produced two distinct bands of labeling in the CNIC, suggesting that the A1-CNIC projection links neurons in both structures with similar frequency tuning (Bajo et al., 2007). This has been confirmed physiologically in the guinea pig by positioning multi-site probes along the tonotopic axes of A1 and the CNIC (Lim and Anderson, 2007). Thus, the activation or inactivation of projections coming from specific cortical frequency bands would shed light about the direct action of A1 neurons on CNIC cells sharing similar tuning properties and reaching similar frequency regions in MGv or CNIC. In addition and regarding A1-MGv projections, Homma et al. (2017) in ferrets have demonstrated mistuning sensitivity in MGv neurons and that feedback from A1 to MGv is required for the normal ability of animals to detect a mistuned harmonic within a complex sound. These studies confirmed the point-to-point connections between the auditory cortex and the subcortical auditory structures.

**FIGURE 3:**
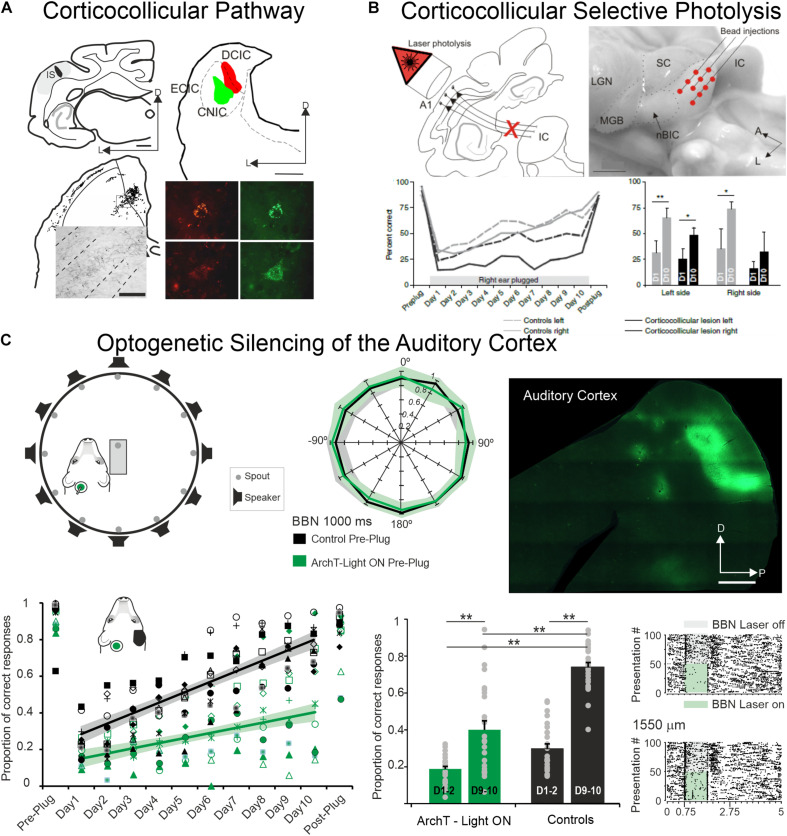
The auditory cortex and the corticocollicular projection are essential for experience-dependent plasticity in spatial hearing. **(A)** Anterograde **(top left)** and retrograde **(top right)** tracer injections in the ferret the auditory cortex and in the Inferior Colliculus, respectively, reveal strong corticocollicular projection with terminal labeled fields in the three IC subdivisions **(bottom left)** after Fluororuby injection in A1 and retrogradely labeled cells in A1 **(bottom right)** after green and red fluorescent retrobead injections in the inferior colliculus. Modified from Bajo et al. (2007). **(B)** Chromophore-targeted laser photolysis of the corticocollicular pathway prevents learning-induced auditory plasticity. Corticocollicular layer V neurons were ablated using an infrared laser light **(top left)** following retrogradely neural labeling after microbead injections in the IC **(top right)**. Percent of correct responses in a sound localization task plotted against days of training including 10 days with unilateral right earplug **(bottom left)**. Data were grouped by left (dashed lines) and right (continuous lines) sound locations with control cases in gray and corticocollicular cases in black. In the bottom right, the mean and SD scores on the first (D1) and tenth day (D10) of monaural earplug are shown. Modified from Bajo et al. (2010). **(C)** Optogenetic silencing of the auditory cortex prevents earplug adaptation but not normal sound localization. Diagram shows the floor plan of the behavioral chamber **(top left)** and sound localization performance (proportion of correct responses at each speaker location). Data from control cases are in black and cases where neural activity in left A1 was optogenetic silenced using ArchT expression and green light illumination during each stimulus presentation in green **(middle panel)**. Histological section of a flattened auditory cortex showing GFP immunofluorescence associated with ArchT expression **(top right)**. Proportion of correct scores averaged across all speaker locations achieved by each animal in the control and A1 silenced groups (preplug session, 10 days with right earplug, and postplug) **(bottom left)**. Proportion of correct responses for the first and last 2 days of monaural occlusion **(middle panel)**. Examples of neural optogenetic suppression in A1 are shown in the bottom right panel. Neural responses driven by broadband stimulation [gray rectangles or combined with laser illumination (green rectangles)]. Modified from Bajo et al. (2019). *P < 0.05, **P < 0.01. Scale bars = 1 mm in **(A,C)**, 2 mm in **(B)**. A, anterior; A1, primary auditory cortex; BBN, broadband noise; CNIC, central nucleus of the inferior colliculus; D, dorsal; DCIC, dorsal cortex of the inferior colliculus; ECIC, external cortex of the inferior colliculus; HP, hippocampus; IC, inferior colliculus; IS, injection site; L, lateral; LGN, lateral geniculate nucleus; MGB, medial geniculate body; nBIC, nucleus of the brachium of the inferior colliculus; P, posterior; SC, superior colliculus.

In a recent experiment in mice using a combination of cortico-anterograde and collicular retrograde viral transfection, it was possible to achieve viral specific transfection of only cortico-collicular neurons (Blackwell et al., 2020). This combination of techniques ensures that only neurons expressing *Cre* recombinase in the auditory cortex would express ChannelRhodopsine2 (ChR2) or a hyperpolarizing opsin (ArchT) in the auditory cortex. Opsins were expressed in AC-IC projecting neurons, and shining light over AC would directly activate, or suppress, only the cortico-collicular feedback projections (Blackwell et al., 2020). ChR2 activation of AC-IC neurons resulted in increasing spontaneous activity in IC neurons with decrease driven activity to pure tones and clicks, but with particularly small effects on magnitude. ArchT silencing of the same pathway has no effect on evoked activity on IC neurons. Both optogenetic manipulations suggest that cortico-collicular feedback does not provide strong modulation on passive listening mice under anesthesia or awake conditions. Consistent with the known cortico-collicular projections, the effects were observed mainly for cells located in the dorsal cortex of the IC (DCIC), not in CNIC. The small reduction in evoked response did not affect the selectivity of IC neurons and did not change the noise correlations during spontaneous and evoked activity. In the same experiment, the authors have tried to determine whether modulating the cortical inhibitory interneurons can change collicular responses (Blackwell et al., 2020). Whereas modulating parvalbumin (PV) interneurons had no effect on spontaneous and tone-evoked activity in IC, suppressing the activity of somatostatin (SST) interneurons increased spontaneous activity in IC. Altogether, this careful study performed both in anesthetized and awake, but passively listening, mice has revealed very little effect of the cortico-collicular projections in such listening conditions.

The main question that can be raised is whether the cortico-feedback projections only exert a strong influence behaving, actively listening, animals. To answer this question, it was necessary to train animals in behavioral tasks and determine the impact of temporary suppression of cortical feedback on behavioral performance.

### Inactivating Specific Auditory Cortex Projections During Challenging Behavioral Tasks

One of the earliest studies that explored the behavioral consequences of suppressing the corticofugal inputs used Elvax implants to release chronically Muscimol, a GABA_a_ agonist (Smith et al., 2004). Ferrets bilaterally implanted with muscimol-Elvax over A1 were trained in a sound localization task with short (40 ms) or long (100–1,000 ms) tone bursts. The implanted animals initially displayed lower correct sound localization during the first sessions, but they improved over time and finally reached the same performance as the control animals. Comparing the silencing of primary and non-primary cortical areas (or making lesions of these areas) induced modest but significant deficits in sound localization and pointed out that the largest deficits were when silencing primary auditory cortex (Nodal et al., 2010, 2012).

In such experiments, the global silencing of the cortex was suppressing all cortical activity not only the feedback to the subcortical structures. To address the question of how cortico-collicular projections impact behavioral responses, two different techniques have been used in the same animal model. First, Bajo et al. (2010, Figure 3B) have used a chromophore-targeted neuronal degeneration technique to investigate the behavioral consequences of selectively eliminating layer V neurons projecting from primary auditory cortical areas to the inferior colliculus. This approach resulted in a loss of about two-thirds of the layer V A1 neurons that project to the IC, without affecting those in surrounding cortical areas or different cortical layers. Most cortico-collicular axons target the ipsilateral IC, so this approach allowed assessing the effects of removing descending axons on one side of the brain, although cross-projections comprise 15% of the cortico-collicular axons that were not eliminated. The behavioral results clearly indicate that ablation of the auditory cortico-collicular pathway from one hemisphere did not affect sound localization, as measured by either the initial orienting response to the sound or the subsequent selection of sound-source location. An interesting challenge was whether the lesioned animals would be able to localize sounds in altered conditions of sound localization such as the one occurring when one ear is occluded and, therefore when the values of binaural cues used for sound localization change (this task was initially described in Kacelnik et al., 2006). While control animals recover their ability to localize sounds accurately with training, despite the continued presence of a plug in one ear, this was not the case in ferrets in which the cortico-collicular projection had been largely removed (Figure 3B), suggesting that descending pathways are essential for recalibration of the brain’s representation of auditory space. This learning deficit was most pronounced in the hemifield contralateral to the lesioned pathway, implying that corticofugal modulation of each IC mediates plasticity in the opposite hemifield (Bajo et al., 2010). Thus, one function of the auditory cortex in spatial hearing is to provide signals that are transmitted via descending cortical pathways to bring about experience-driven changes in localization.

Second, silencing auditory cortex neurons (by light stimulation of neurons expressing the proton pump ArchT) during sound presentations in an azimuthal sound-localization task did not impair the initial animals’ behavioral performance (Bajo et al., 2019, Figure 3C): performance of control animals and the animals in which each stimulus presentation was paired with optogenetic silencing of A1 neurons localized broadband noise bursts was equally similar (Figure 1 in Bajo et al., 2019). When the animals were trained to re-learn the sound-localization task after unilateral ear occlusion (after plugging one ear), there was a massive drop in performance both in controls and in animals with optogenetic control of A1. Nonetheless, across 10 days of training to perform the task with monaural occlusion (note that plugging one ear change the values of the binaural cues but do not eliminate binaural cues), the control animals considerably improved their performance which was not the case for the animals for which A1 was silenced during each trial during sound delivery (Figure 4 in Bajo et al., 2019, Figure 3C). Thus, suppressing auditory cortex activity did not prevent the animal to normally localized sounds, but impaired the ability to adapt to a unilateral earplug.

**FIGURE 4:**
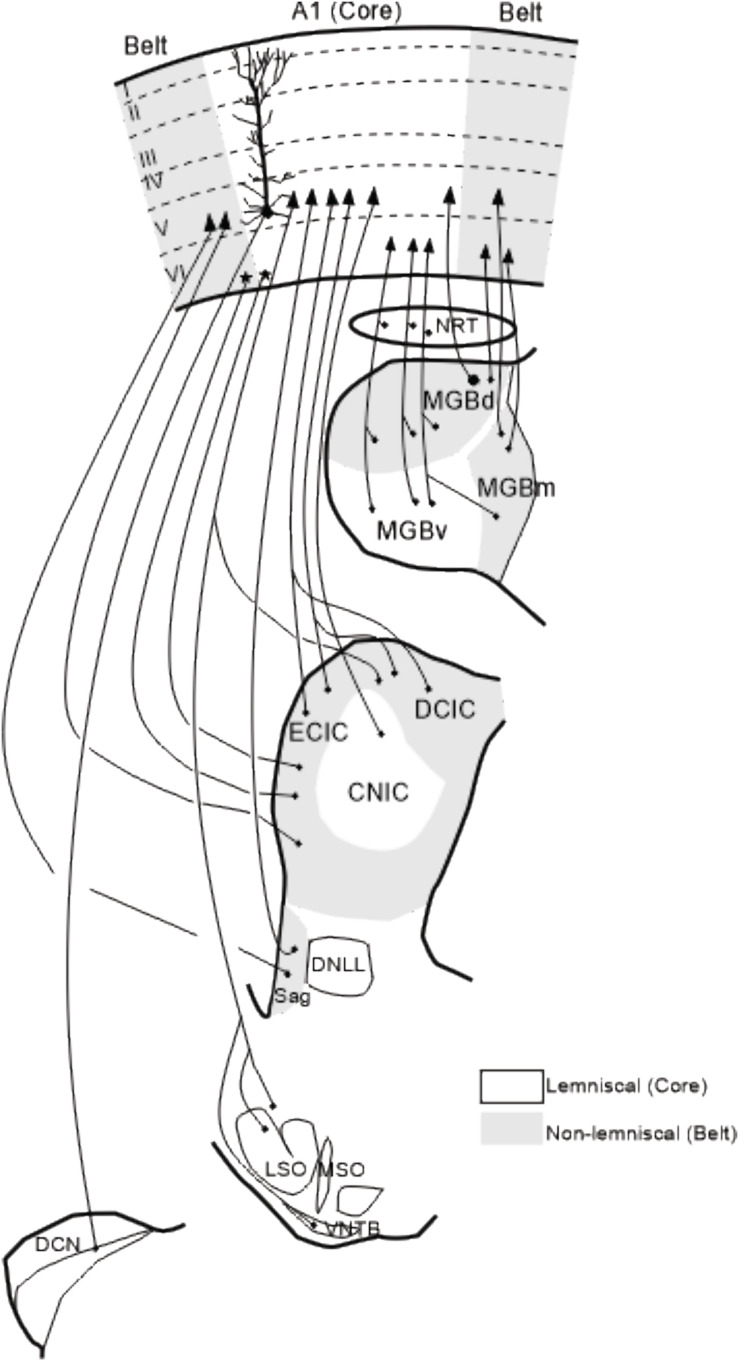
Summary of corticofugal projections to auditory nuclei. Corticofugal projections to the nucleus reticularis thalamic, auditory thalamus and Sagulum are ipsilateral whereas projections to the inferior colliculus, superior olivary complex and cochlear nucleus are bilateral although predominantly from the same side. Modified from Winer (1992) and Malmierca et al. (2015). The details of these corticofugal projections are described in the following papers: Beyerl (1978); Adams (1980); Druga and Syka (1984a, b); Faye-Lund (1985); Feliciano and Potashner (1995); Saldaña et al. (1996); Weedman and Ryugo (1996); Beneyto et al. (1998); Winer et al. (1998); Winer et al. (2001); Jacomme et al. (2003); Bajo and Moore (2005); Meltzer and Ryugo (2006); Bajo et al. (2007); Coomes Peterson and Schofield (2007); Schofield (2009); Saldaña (2015); Lesicko and Llano (2017). A1, primary auditory cortex; CNIC, central nucleus of the inferior colliculus; DCN, dorsal cochlear nucleus; DCIC, dorsal cortex of the inferior colliculus; DNLL, dorsal nucleus of the lateral lemniscus; ECIC, external cortex of the inferior colliculus; LSO, lateral superior olive, MGBd, dorsal division of the medial geniculate body; MGBm, medial division of MGB; MGBv, ventral division of MGB; MSO, medial superior olive; NRT, nucleus reticularis of the thalamus; Sag, nucleus sagulum; VNTB, ventral nucleus of the trapezoid body; I–VI, cortical layers 1–6.

An additional surprising finding was observed when the same ear was occluded for a second time in control animals that had previously adapted to the unilateral hearing loss. A much smaller initial deficit was observed when the ear was replugged than when the animals first experienced an earplug. Furthermore, most of the control ferrets achieved their maximum score by ∼day 5 and remained at around that level until the end of the second period of monaural occlusion. In contrast, the ArchT animals (that had previously shown impaired adaptation when cortical activity was suppressed) re-tested with the occluded ear but *without silencing the auditory cortex* did not show a better adaptation than during the first earplug. Despite a normal activity in the auditory cortex, these animals adapted at the same rate as that observed during the first period of monaural deprivation when A1 was inactivated, and significantly more slowly than the control animals during their first period of monaural occlusion. Thus, optogenetic suppression of cortical activity not only impairs auditory spatial learning, but also results in less effective adaptation when the active auditory cortex is subsequently challenged by monaural occlusion. When the auditory cortex was again inactivated on these animals, their performance was exactly the same as with the cortex intact, suggesting that the limited capacity of these animals to adapt to the second period of monaural occlusion no longer appears to be dependent on the activity of A1 (**Figure 6** of Bajo et al., 2019).

Both examples show the relevance of the auditory cortex and of the cortico-collicular projections in actively listening animals performing challenging behavior tasks.

## Deciphering the Mechanisms Underlying the Corticofugal Effects

Corticofugal projections are particular abundant in the auditory system (Figure 4; Winer, 2006). An important concept that has been proposed for understanding the functional role of corticofugal projections within the thalamo-cortical sensory systems is the distinction between “driver” and “modulator” inputs (Sherman and Guillery, 1998, 2002; Guillery and Sherman, 2002) which have been re-named Class 1 and Class 2 inputs (Lee and Sherman, 2010, 2011) based on the initial anatomical description by Guillery (1966). In the auditory system, this distinction leads to the possibility that the cortical afferents from A1 reaching MGv are modulatory inputs for the lemniscal (MGv) relay cells (review in Lee and Sherman, 2010, 2011). Note also that the impact of the cortical inputs can also be indirect via the thalamic reticular nucleus (TRN), which can have a stronger influence on the lemniscal division than on the non-lemniscal ones (cats: Crabtree, 1998; rats: Cotillon-Williams et al., 2008).

From the previous section, it seems that the crucial point that needs to be explored is how a cortical input projecting on IC cells (or MGv cells) which is, in some contexts, a modulator that modestly affects the functional properties of IC cells in awake passive animals (Blackwell et al., 2020) becomes a necessary input that can be used to drive the animal behavioral response (Bajo et al., 2010, 2019). In other words, what are the factors that, surprisingly, transform a potential weak and inefficient cortico-collicular input into a driving force that can guide the animal in its behavior? Could corticofugal projections act as drivers or modulators in a context dependent manner? The next question is how the subcortical networks are affected by cortical inputs depending on the difficulty of the task and the stability of those changes in time.

Here, we consider that in anesthetized animals and in awake animals that are not engaged in a behavioral challenging task, the corticofugal descending projections are only parts of the synaptic excitatory inputs reaching thalamic and collicular cells. In contrast, we would like to propose that the auditory corticofugal projections play an essential role during active listening associated to challenging behavioral tasks, under the dual control of neuromodulatory systems and the frontal cortical areas.

### Neuromodulation in the Auditory Cortex

The most obvious factor that can change the way auditory stimuli are processed in awake animals between “passive” vs. “actively listening” conditions is the involvement of the neuromodulatory systems. Among them, the noradrenergic, dopaminergic and cholinergic systems have long been implicated in behavioral situations and cognitive functions (noradrenergic: Sara, 2009; dopaminergic: Seamans and Yang, 2004; Wise, 2004; Schultz, 2016; Ott and Nieder, 2019; cholinergic: Sarter et al., 2005; Lin et al., 2015). Two main properties should be considered about these neuromodulatory systems.

First, all brain nuclei at the origin of these neuromodulators are engaged, at different degrees, in cognitive functions. For example, neurons in the locus coeruleus (LC), the cortical source of noradrenaline (NA), are responsive to stimuli of any modality associated with reinforcements (Sara and Segal, 1991; Aston-Jones et al., 1997; Bouret and Sara, 2004). Dopaminergic neurons of the ventral tegmental area (VTA) are activated by rewards, and code for specific aspects of rewards such as their amount, probability of occurrence, subjective value, as well as to any reward-predicting stimuli, and their level of prediction of the reward occurrence (reviewed in Schultz, 2016). The cholinergic inputs arising from the basal forebrain (BF) area has long been involved in learning, acquired-stimulus salience and more generally in all situations of “attentional effort” (Sarter et al., 2006). In addition, experience dependent adaptation to the altered binaural cues was disrupted after the cortical cholinergic depletion in ferrets (Leach et al., 2013).

Needless to say, these three neuromodulatory systems do not work independently of each others, they all work in concert for controlling the state of cortical arousal and allowing cognitive performance. In fact, both in cortical and subcortical structures, non-synaptic interactions occurring at the presynaptic level are common and lead to subtle regulations of the excitatory and inhibitory transmission by a synergy between neuromodulators (reviewed in Vizi and Lábos, 1991; Vizi et al., 2010; Sperlágh and Vizi, 2011).

More importantly, these three neuromodulators drastically modify the processing of acoustic stimuli in the auditory cortex, and more generally, in the entire auditory system. For example, in guinea pigs, iontophoretic applications of NA increase the sharpness of tuning of auditory cortex neurons (Manunta and Edeline, 1997, 1998, 1999) and the neuronal discrimination performance between conspecific vocalizations (Gaucher and Edeline, 2015). Acetylcholine has a dual action on auditory cortex neurons. Whereas some effects were attributed to muscarinic receptors (mAChR; Guinea pigs: Metherate et al., 1990; Mice: Chen and Yan, 2007; Rats: Froemke et al., 2007), other studies proposed that the action of nicotinic receptors (nAChR) was prominent (Mice: Kawai et al., 2007; Rats: Liang et al., 2006). In fact, activation of mAChRs tends to increase postsynaptic excitability while decreasing intracortical transmission via presynaptic receptors, whereas, in contrast, activation of nAChRs enhances thalamocortical transmission (reviewed in Edeline, 2003; Metherate, 2011). Only a few studies have described the dopaminergic modulation in the auditory cortex. In monkeys, it was shown that electrical stimulation of VTA modifies neuronal activity in the auditory cortex on two time scales: (i) effects on the time scale of tens to hundreds of milliseconds (Macaque monkeys: Mylius et al., 2015), and (ii) effect on the time scale of seconds and minutes that were reflected in the spontaneous and evoked activity (Huang et al., 2016). In gerbils, systemic administration of D1/D5 dopamine receptor agonists enhanced early infragranular auditory-evoked synaptic activity, prolonged auditory cortex activation, and more effectively recruited horizontal corticocortical networks during later phases of evoked activity (Happel et al., 2014). Note that neuromodulators alter auditory processing before the cortical level: Dopamine modulates the processing of unexpected auditory information as early as the inferior colliculus (Rats: Valdés-Baizabal et al., 2020), locus coeruleus activation alters thalamic and cortical responses to the same extent (Guinea pigs: Edeline et al., 2011), the pontomesencephalic cholinergic system modulates the activity of auditory thalamus and inferior colliculus (Woolf, 1991; Guinea pigs: Schofield et al., 2011), and NA modulates the response strength and the response latency as early as the cochlear nucleus (Mustached bat: Kössl and Vater, 1989) and by its action on the olivo-cochlear neurons can also modulate the compound action potential (Guinea pig: Mulders and Robertson, 2005a, b).

Although not historically considered major modulators of cortical processing, neuropeptides and neurohormones are now considered as such. For example, growing evidence suggests that oxytocin (OT) acts to enhance the salience of socially relevant sensory inputs and is important for parental behavior and social cognition. This peptide is synthesized in the paraventricular nucleus and supraoptic nucleus of the hypothalamus and binds to a G protein–coupled receptor with a single isoform (Gimpl and Fahrenholz, 2001). A series of studies have looked into the role of oxytocin in maternal behavior and in the processing of ultrasonic vocalization of pups when separated from the nest. Some studies have not found enhanced responses to pup calls between virgin and mother mice (Liu and Schreiner, 2007; Shepard et al., 2016; Royer et al., 2021). However, pharmacological application of oxytocin or optogenetic release of OT on the left auditory cortex (Marlin et al., 2015) reduced call-evoked inhibitory post-synaptic potentials (IPSCs) within seconds (**Figures 6A,B**, open and filled symbols respectively, and extended data **Figure 8** in Marlin et al., 2015), whereas the excitatory post-synaptic potentials (EPSCs) were gradually modified over minutes (**Figures 6A,B**, filled in Marlin et al., 2015). Therefore, oxytocin seems to rapidly disinhibit the auditory cortex (potentially similarly to ACh), suggesting that it can regulate attention and increase the salience of social stimuli. These results corroborate the effects of oxytocin in hippocampal slices (Rats: Owen et al., 2013).

Similar to oxytocin, orexins (Orexin A and B) are neuropeptides that profusely innervate the brain, including the deep layers of the neocortex (Marcus et al., 2001), and modulate the action of other classic neuromodulators (Peyron et al., 1998; but see Flores et al., 2015 for review). The orexin system is comprised of a small population of cells located mainly in the lateral hypothalamus. Orexins bind to specific receptors (OX1R and OX2R), associated with a Gq protein that activates the phospholipase C–protein kinase C pathway producing neural depolarization and increasing the membrane resistance by the closure of the K^+^ conductance. Functions of the orexin system include the modulation of arousal and sleep–wake cycles, energy homeostasis, reward processing, stress and emotional behavior regulation (for example modulation of fear memory). Orexin might directly affect the auditory corticofugal pathways thanks to the specific expression of its receptors in layers V and VI (Rats: Marcus et al., 2001). In somatosensory and visual cortices, orexins induce functional changes in layer VIb neurons (Rats: Bayer et al., 2002). Layer VIb auditory neurons project to the inferior colliculus (Cats: Winer et al., 1998; Gerbils: Bajo and Moore, 2005; Guinea pigs: Schofield, 2009). In addition, the effect of the orexins might be indirectly mediated by the activation of the non-specific thalamocortical projections from the intrathalamic and midline nuclei (Bayer et al., 2002). Other indirect pathways might involve the medial prefrontal cortex (mPFC) cholinergic basal forebrain and locus coeruleus that show a great expression of orexin receptors (Marcus et al., 2001) and are capable as discussed above, of modulating auditory processing.

These studies indicate that in addition to the classical neuromodulators, oxytocin and orexins are also key actors to modulate the action of the cortical descending pathways.

### Implications of the Frontal Areas in Attentional Processes During Active Auditory Listening

Attention is vital to achieve goals in constantly changing sensory environments. Frontal areas have long been suspected to play an important role in attentional processes. In primates, the auditory cortex projects and receives influence of higher order areas in the frontal cortex (Hackett et al., 1999; Romanski et al., 1999; Romanski and Averbeck, 2009). Over the last decades, electrophysiological recordings combined with behavioral tasks have demonstrated on one hand that, correlations of neuronal activity exist between the auditory cortex and frontal areas and, on the other hand, that there are also causal links between these two regions. Indeed, during tone detection tasks, Fritz et al. (2010) in ferrets showed that the activity of frontal cortex neurons was modulated by task events, but either by increasing or suppressing their firing rate to the target stimuli. In contrast, they lost responsiveness to identical stimuli presented passively, suggesting that frontal responses are tightly linked with the behavior. However, in these experiments, only a weak correlation between target response strength and task performance was observed. When the task was performed with visual cues, about one-third of the responsive frontal cells showed responses to both auditory and visual targets with similar responses to the two sensory modalities. The unimodal cells however presented different responses suggesting that some frontal cortex responses are modality specific. Interestingly, coherence analysis of local field potential (LFP) signals simultaneously recorded in A1 and frontal cortex showed that during active behavior, the synchronous activity between these areas is selectively enhanced when the target stimuli are presented but attenuated for responses to the reference sound. They argued that when an animal is engaged in a behavior, attention enhanced the synchronous activity between A1 and the frontal cortex.

To go further, Atiani et al. (2014) in the same animal model, compared responses obtained in A1, in two cortical belt areas and in dorsolateral frontal cortex during the same auditory discrimination task as Fritz et al. (2010). They showed that contrast enhancement between target and reference responses becomes more pronounced in frontal cortex than in auditory belt areas and than in A1. Thus, the reference responses are gradually suppressed as signals are transmitted through higher-order areas to frontal areas. In fact, recent analyses suggest that the neuronal responses became more categorical in higher cortical areas during task performance (Yin et al., 2020). Overall, these studies pointed out strong relationships between the activity in frontal and auditory cortex when an animal is engaged in an auditory discrimination task (Fritz et al., 2003, 2010; Atiani et al., 2014).

In primates, very few studies have investigated frontal cortical activity in various auditory behaviors to reveal the specific cognitive functions as decision making or reward value, associated with the network frontal cortex-auditory cortex. Tsunada et al. (2019) recorded neural activity from the ventrolateral prefrontal cortex (vlPFC) in two monkeys in a frequency discrimination task where they have to determine whether the tone bursts were predominantly “‘low frequency”’ or “‘high frequency.” They showed that post-decision vlPFC activity encodes the key features of the previous completed decision process that are used to generate the next one. Electrical microstimulation at vlPFC sites affected the monkeys’ choices on the subsequent, but not the current, trial confirming that vlPFC activity is related to the encoding of the past trials and also informative in subsequent trials (for review, Banno et al., 2020).

Recently, Huang and Brosch (2020) recorded neuronal activity from vlFC in parallel with the neuronal activity from the auditory cortex of a single monkey performing two go-no go behavioral tasks requiring different audiomotor associations and using a sequence of two tones. Interestingly, they showed that, in the auditory cortex, the representations of the two tones were related to behavior. In contrast, in PFC, such a behavioral relevance was observed only for the first tone of the sequence. They thus promote the idea that the audiomotor representations in AC were more strongly related to behavior than those in PFC.

But does the activity in frontal areas provide enough excitatory inputs to drive auditory cortical neurons? Some studies used targeted-stimulation methodologies for demonstrating the relationships between neuronal activity in the frontal cortex and its effect in the auditory cortex in mice (Winkowski et al., 2013, 2018). First, the authors investigated the orbitofrontal cortex (OFC) stimulation on the neuronal activity in A1 using two-photon calcium imaging technique in mice (Winkowski et al., 2013). They found a diversity of effects, but often after pairing a particular frequency with the electric stimulation of OFC, the best frequency of A1 neurons in layers II/III changed with a response enhancement near the particular frequency used. Their results suggest that OFC activation could regulate neuronal activity within A1. Optogenetic activation of the mouse OFC in an area where neurons respond to sounds, activate A1 neurons and current source density (CSD) analysis revealed current sinks in layer I and layer IV, providing activation to both pyramidal cells and interneurons (Winkowski et al., 2018).

Last, in a recent study, Olthof et al. (2019) described in adult rats, that the inferior colliculus, receives dense descending projections not only, from the auditory cortex, but also from the visual, somatosensory, motor, and prefrontal areas suggesting that the inferior colliculus can also integrate information coming from higher cortical areas.

## Possible Scenarios

**FIGURE 5:**
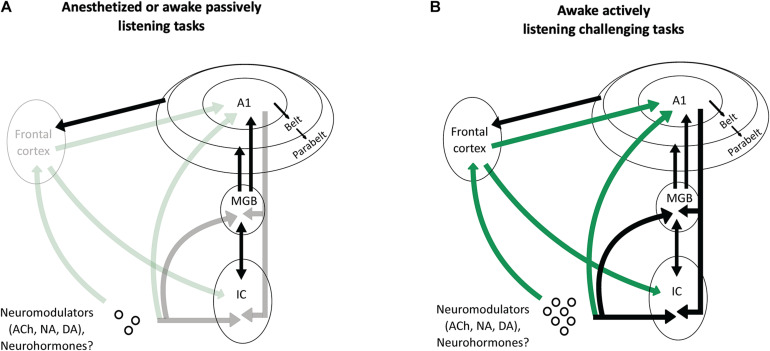
Potential scenarios involving frontal projections and neuromodulators as key factors to increase the impact of the cortical descending projections on the thalamus and the inferior colliculus. **(A)** In anesthetized animals, or in awake passively listening animals performing easy auditory tasks, there is no or little activation of the frontal areas and of the neuromodulatory systems (symbolized by the shaded arrows). As a consequence, the descending projections from auditory cortex have a negligible effect on the processing of acoustic information by subcortical auditory structures (MGB and IC). **(B)** In awake actively listening animals performing challenging tasks, there is a strong activation of the frontal cortical areas and neuromodulatory systems (symbolized by the solid green lines). This activation sends a strong input to modulate the activity of the auditory cortex, which in turn, reshape the subcortical network (MGB and IC) thus allowing to perform successfully during this task.

Based on the recent findings presented above, we propose that one of the fundamental roles of the frontal cortical areas and the neuromodulatory systems is to increase the efficacy that the cortical descending pathways exert on the subcortical structures when an animal is performing a challenging complex auditory task (Figure 5).

In anesthetized or awake passive conditions, or during basic auditory tasks, the descending projections from auditory cortex to subcortical structures are probably not necessary: in those cases, when the level of attention is absent (under anesthesia) or low (under passive listening), the frontal cortex and the neuromodulatory systems send no or little information to the cortical auditory areas. Under these conditions, the descending cortical inputs represent only a fraction of the excitatory inputs that subcortical neurons can use to build robust representations of the auditory scene. In contrast, when the task becomes challenging and the attentional level increases, the frontal areas and neuromodulatory systems are strongly activated (Humans: Berry et al., 2015; Du et al., 2016; Dimitrijevic et al., 2019; Monkeys: Lecas, 1995; reviewed in Peelle, 2018) and these inputs drastically change the activity of auditory cortex neurons. As a consequence, the descending cortical inputs to the subcortical auditory structures (MGB, IC, or even dorsal cochlear nucleus) send crucial information about the most adapted behavioral response needed to perform succesfully in these difficult conditions. In the case of the experiments discussed in this review, challenging conditions could be, for example, when an animal is engaged in discrimination tasks with noisy stimuli at very low SNRs, or during sound localization with an occluded ear.

Two possibilities can be envisioned for the emergence of the behavioral meaning at the subcortical level. Either the cortical descending inputs allow subcortical structures to generate more robust representations by plasticity mechanisms operating in the subcortical networks.

Alternatively, cortical input maximally account for the information already present at the subcortical level to perform the behaviorally challenging task. Collecting subcortical electrophysiological recordings during these challenging tasks with and without suppressing the descending cortical projections is probably the only way to determine which of these two assumptions is valid.

It is important to determine what are the relative contributions of the inputs from the frontal areas and from the neuromodulators. For example, data in humans suggest that subjects with a polymorphism of the choline transporter gene that is thought to limit choline transport capacity (Ile89Val variant of the choline transporter gene SLC5A7, rs1013940) do not show a robust activation of the right prefrontal cortex (Brodmann’s areas 9) during challenging attentional tasks, whereas control subjects do (Berry et al., 2015). In addition, it is important to point out that the neuromodulators do not impact only the cortical level but also the subcortical structures, and that a single neuromodulator such as noradrenaline can influence auditory responses from cochlear nucleus (Mustached bats: Kössl and Vater, 1989) up to auditory cortex (Guinea pigs: Manunta and Edeline, 1997; Edeline, 1999; Gaucher and Edeline, 2015). Additionally, an important parameter that should be explored in the future is the timing of the network activation leading the animal to successful performance in a challenging task, and the stability of the network activation related to learning. A disruption in the network synchronization, or a delay in the activation of a key structure (as the frontal cortex, auditory cortex or in the release of some neuromodulators) could also contribute to behavior failure.

This dual control allows the auditory cortex to instruct subcortical structures about the meaning of each stimulus, its relationships with rewards, and the exact nature of the behavioral/motor response that need to be applied at the occurrence of a given stimulus in a particular environment. Although speculative, this scenario should be tested in future experiments.

## Author Contributions

SS and J-ME wrote the initial draft of the review. VMB and FRN revised the manuscript for important intellectual content and approved all aspects of the review. SS, VMB, and FRN designed the figures of the review. All authors contributed to the article and approved the submitted version.

## Conflict of Interest

The authors declare that the research was conducted in the absence of any commercial or financial relationships that could be construed as a potential conflict of interest.

## Publisher’s Note

All claims expressed in this article are solely those of the authors and do not necessarily represent those of their affiliated organizations, or those of the publisher, the editors and the reviewers. Any product that may be evaluated in this article, or claim that may be made by its manufacturer, is not guaranteed or endorsed by the publisher.
